# Digital exposure and health behaviours in adolescents: a cross-sectional study from South India

**DOI:** 10.3389/fped.2026.1883252

**Published:** 2026-07-29

**Authors:** Sharlet Dsouza, Supreetha Mendon

**Affiliations:** Nitte (Deemed to be University), Nitte Usha Institute of Nursing Sciences (NUINS), Department of Child Health Nursing, Mangalore, India

**Keywords:** adolescence, diet, health promotion, physical activity, screen time

## Abstract

**Background/objectives:**

Adolescence is a critical developmental period during which lifestyle behaviours such as diet, physical activity, and screen use are established. Increasing screen exposure and changing lifestyle patterns have raised concerns regarding their impact on health outcomes. This study aimed to assess eating behaviour, physical activity, and screen time among adolescents and examine their association with demographic factors in a school-based setting in South India.

**Methods:**

A cross-sectional descriptive study was conducted among adolescents aged 12–15 years using a purposive sampling technique. Data were collected using structured questionnaires, including a sociodemographic proforma, Johnson Adolescent Food Habits Checklist, Physical Activity Questionnaire, and Questionnaire for Screen Time (QueST). Statistical analysis was performed using SPSS version 29.0, and associations were assessed using chi-square tests with significance set at *p*-value < 0.050.

**Results:**

Total of 192 adolescents were included. Most adolescents demonstrated neutral eating behaviour (93.2%). Physical activity classification revealed that 54.7% of participants were highly active, 44.8% reported low physical activity, and 0.5% (*n* = 1) were moderately active. Screen exposure was reported across academic and recreational domains. Across the screen-time domains, 14.1%–20.3% reported screen exposure of 2 h or more per day. Significant associations were observed between physical activity and sex (*p*-value = 0.006), family type (*p*-value = 0.015), exercise status (*p*-value = 0.018), and age (*p*-value < 0.050). Eating behaviour was significantly associated only with type of food (*p*-value = 0.029). Screen time showed no significant association with most demographic variables, except for age, which was significantly associated with increased screen exposure in specific domains.

**Conclusions:**

This study found that most adolescents had eating-behaviour scores in the intermediate range, physical activity levels were mainly divided between low and high activity, while screen use was reported across both academic and recreational domains, with 14.1%–20.3% of adolescents reporting in excess of two hours per day. Physical activity was significantly associated with sex, family type, exercise status, and age, whereas eating behaviour was associated with type of food. Screen time was associated with age only in the studying and entertainment domains. Collectively, these findings elucidate the need for school-based health promotion strategies, encompassing regular physical activity programmes, nutrition education oriented towards balanced meal routines, and age-sensitive screen-time management guidance addressing both academic and recreational digital engagement.

## 1 Introduction

The trajectory between onset of puberty to end of adolescence involves immense changes in an individual's biological, cognitive, psychosocial, and emotional development states (1). These significantly influence lifestyle behaviours, which in turn play a major role in shaping overall health outcomes (2). Beyond hormonal changes, rapid growth spurts, and heightened energy requirements, adolescent dietary patterns are also strongly influenced by external environmental and social determinants (2). Peer norms, shifting social behaviours, and the widespread availability of low-cost convenience foods contribute to increasingly unhealthy food choices (3). In addition, pervasive youth-oriented marketing of sugar sweetened beverages, packaged snacks, and other ultra processed products further shapes dietary preferences toward calorie dense, nutrient poor diets during this critical developmental period (4).

Driven by a cluster of lifestyle factors including sedentary activities such as increased screen time, transitioning food and sleep habits as well as abundance of unhealthy options have propelled the prevalence of high BMI among adolescents aged 10–19 years (5, 6). Obesity or overweight in adolescents has risen from about 8.4% in 1990 to around 17.6% in 2021, marking consistent sustained upward trend over the past three decades (7). Effective obesity prevention strategies for contemporary adolescents, who are exposed to pervasive digital food marketing, necessitate the integration of targeted interventions to regulate digital device use alongside the promotion of health-enhancing dietary patterns and physical activity behaviours.

According to UNICEF, 49% of teenagers in high-income countries eat fast food once a week, while 42% of adolescents in low- and middle-income countries drink sugary sodas every day (8). Adolescents themselves report that screen time frequently triggers impulsive and compulsive consumption of UPFs, particularly snacks, through pervasive food advertising and easy availability of such products (7). A study in a western India setting reveals an alarmingly high prevalence of excess screen time at 83.2% among rural Indian adolescents, with a significant impact on sleep patterns (*p* = 0.001), highlighting an urgent need for intervention (8). Screen exposure often operates at a subconscious level, reinforcing unhealthy dietary habits by normalising energy dense, nutrient poor foods in advertisements and entertainment settings (9, 25). This evidence supports the idea that screen behaviours and dietary risks cluster together, contributing to the obesity epidemic and other metabolic risks in adolescents (10).

Adolescents should adopt healthy eating habits to prevent long-term health issues, as unhealthy diet is linked to chronic disease (11, 12). Promoting a balanced lifestyle characterised by healthy dietary practices, adequate physical activity, and limited recreational screen exposure is increasingly recognised as essential for safeguarding adolescent health and preventing the early onset of non-communicable disease (NCD) risk (13). As adolescence is a critical age period for forming lifelong habits related to nutrition, exercise, rest and performing activities of daily living which significantly impact short- and long-term health outcomes (11).

In India, adolescents constitute 20% of the population and the concerning trends in consumption of digital media and unhealthy diet, contributes to the “double burden” of malnutrition/undernutrition alongside rising overweight/obesity (14). Despite the importance of this developmental period, context specific evidence on these interrelated lifestyle behaviours remains limited (14). It is important to explore the impact of these interconnected behaviours in the South Indian context as these are modifiable risk factors for chronic conditions like cancer, diabetes, and heart disease. By evaluating these in a real-world school setting, the study provides actionable insights for interventions to prevent NCD escalation, aligning with the document's emphasis on understanding these connections to “reduce the risks and improve overall population health”.

The present study aimed to assess eating behaviour, physical activity patterns, and screen time among adolescents aged 12–15 years in selected schools in Mangalore. Additionally, it seeks to examine the associations between these behavioural factors and selected demographic variables, thereby generating evidence to inform targeted school-based health promotion strategies and early preventive interventions.

## 2 Materials and methods

### 2.1 Study design and population

This study employed a cross-sectional descriptive design to assess eating behaviour, physical activity, and screen time among adolescents. The study population comprised adolescents aged 12–15 years enrolled in tertiary schools and available during the data collection period were included. The age range of 12–15 years was selected as this represents early-to-mid adolescence, a developmental period during which lifestyle behaviours related to diet, physical activity, and screen use are actively being established and are particularly amenable to health promotion interventions.

While those with known medical conditions were excluded. This study used a purposive sampling technique. The data was collected from July 2024 to August 2024. The sample size was calculated using OpenEpi Version 3, open-source calculator for sample size for frequency in a population. The sample size was calculated assuming a prevalence of 56.9% based on a previous cross-sectional study among Indian adolescents (11). An absolute allowable error of 5%, 80% confidence interval, and 10% non-response rate. The minimum required sample size was estimated to be 178 participants. A total of 192 adolescents were included in the final analysis.

### 2.2 Data collection procedure

Ethical clearance was obtained from the Institutional Ethics Committee. Formal written permission was obtained from the respective school authorities. Prior to enrolment, written informed assent and informed consent was obtained from the participant and parent or legal guardian of each participant respectively. Confidentiality of all responses was maintained throughout the study. Data were collected using structured questionnaires administered to participants in the school setting. The sociodemographic proforma and study tools were administered in a single session.

### 2.3 Study instruments

We established content validity of the tools through expert review by five specialists from relevant disciplines. Necessary modifications were made based on their feedback. The pilot study was conducted among 20 adolescents aged 12–15 years from the target population to assess feasibility, comprehension, and reliability. Data were collected using four tools:

Sociodemographic Proforma: A structured questionnaire consisting of 10 items assessing baseline characteristics such as age, sex, family type, dietary habits, exercise patterns, and screen usage.

Adolescent Food Habits Checklist (AFHC): Healthy eating behaviour was assessed using the 23-item self-report AFHC developed by Johnson, Wardle and Griffith (2002) with good convergent validity, internal consistency and test-retest reliability (15). Each item is scored so that a healthy response receives one point, with ‘not applicable’ and missing responses adjusted using the standard correction; higher total scores indicate healthier eating behaviour. The AFHC has been used in previous Indian adolescent study however, it has not been formally validated or culturally adapted specifically for Indian adolescents, and no India-specific Cronbach's alpha was identified. In the present pilot study, the AFHC showed an internal consistency of Cronbach's *α* = 0.60 (95% CI: 0.29–0.81). For the purpose of description and interpretation, the AFHC scores were categorised using the following cut offs: unhealthy, ≤9; neutral, 10–14; and healthy, ≥15. The neutral category represented scores between the unhealthy and healthy thresholds, indicating neither clearly healthy nor clearly unhealthy eating behaviour.Children's Physical Activity Questionnaire (cPAQ): Physical activity was assessed using the 11-item Children's Physical Activity Questionnaire (cPAQ), a self-administered instrument developed to assess habitual physical activity among school-aged children. Responses were categorised into low, moderate and high activity for description (16). In a validation study involving 90 children aged 9–10 years, the cPAQ demonstrated moderate-to-high test-retest reliability (*r* = 0.55–0.68, *p* < 0.001) and reasonable validity for moderate-intensity activity when compared with accelerometer-derived measures (17). While most of the questionnaire is in five-point scale formats with higher scores indicating greater physical activity, there were questions to assess if the measured week was typical and these were excluded from scores.

Questionnaire for Screen Time (QueST): Screen time assessment was done using a 5-item questionnaire which measures self-reported screen exposure across five domains: studying, watching television or videos, playing video games, and social media or chat application use. It has demonstrated satisfactory content validity and fair-to-good test-retest reliability among adolescents (18). For each domain, the adolescents reported their usual screen-time duration on a typical day using four response categories: less than 1 h, 1–2 h, 2–3 h, and more than 3 h. Each domain was analysed separately because academic, recreational, gaming, and social media screen use represent distinct behavioural contexts.

### 2.4 Statistical analysis

Data were coded and entered into a master sheet for analysis. Statistical analysis was performed using SPSS version 29.0. Categorical variables were expressed as frequency and percentage. Associations between categorical variables were examined using the Chi-Square (*χ*^2^) test. Where the assumptions of the Chi-Square test were violated i.e., specifically when expected cell frequencies were less than 5 Fisher's Exact Test was applied. Statistical significance was set at *p*-value < 0.050.

## 3 Results

### 3.1 Participant characteristics

A total of 192 adolescents were included in the analysis. The baseline characteristics are presented in Table 1.

**Table 1:** Demographic of variables of the adolescents.

Baseline variable		*n*	%
Age	12–<13	11	5.7
	13–<14	54	28.1
	14–<15	127	66.1
Sex	Female	95	49.5
	Male	97	50.5
	Total	192	99.0
Place of living	Hostel	8	4.2
	Home	184	95.8
	Total	192	100
Type of family	Nuclear	119	61.9
	Joint	73	38
	Total	192	100
Food intake	Once a day	10	5.2
	Twice a day	23	12.0
	Thrice a day	146	76
	Four times a day	13	6.8
	Total	192	100
Type of food	Vegetarian	71	37
	Non-vegetarian	121	63
	Total	192	100
Exercise	Yes	116	60.4
	No	76	39.6
	Total	192	100
Duration of exercise	30–<45 min	126	65.6
	45 to <60 min	18	9.4
	60 to <120 min	10	5.2
	120 to <180 min	7	3.6
	None of the above	31	16.1
	Total	192	100
Mobile phone usage	Yes	172	89.6
	No	20	10.4
	Total	192	100
Time spent in mobile	30–<60 min	120	62.5
	60–<120 min	57	29.7
	120–<180 min	9	4.7
	≥180 min	6	3.1
	Total	192	100

The majority of participants were aged 14–15 years (66.1%), followed by 13–14 years (28.1%) and 12–13 years (5.7%). The sex distribution was nearly equal, with 50.5% males (*n* = 97) and 49.5% females (*n* = 95). Most participants were day scholars (95.8%) and belonged to nuclear families (61.9%).

Regarding lifestyle characteristics, 76% of adolescents reported consuming three meals per day, and 63% were non-vegetarian. Approximately 60.4% reported engaging in regular exercise. Among all participants, 65.6% reported exercising for 30–< 45 min. Mobile phone usage was highly prevalent (89.6%), with most adolescents (62.5%) reporting screen exposure between 30 and 60 min daily.

### 3.2 Eating behaviour, physical activity, and screen time

The distribution of eating behaviour, physical activity, and screen time is presented in Table 2.

**Table 2:** Assessment of eating behaviour, physical activity and screen time among adolescents.

Variable		*n*	%
Eating behaviour/habit.	Unhealthy	2	1.0
	Neutral	179	93.2
	Healthy	11	5.7
Physical activity	low physical activity	86	44.8
	moderate physical activity	1	0.5
	high physical activity	105	54.7
Screen time			
Studying on computer or electronic devices	<1 h	98	51.0
	1 to <2 h	67	34.9
	2 to <3 h	18	9.4
	≥3 h	9	4.7
Schoolwork on tablet, smartphone.	<1 h	95	49.5
	1 to <2 h	66	34.4
	2 to <3 h	13	6.8
	≥3 h	18	9.4
Entertainment watch TV show, movies, cartoon or other devices.	<1 h	109	56.8
	1 to <2 h	48	25.0
	2 to <3 h	28	14.6
	≥3 h	7	3.6
Playing video games	<1 h	115	59.9
	1 to <2 h	48	25.0
	2 to <3 h	16	8.3
	≥3 h	13	6.8
Social media like Facebook, Instagram, snapchat, WhatsApp, telegram or other devices.	<1 h	108	56.3
	1 to <2 h	45	23.4
	2 to <3 h	21	10.9
	≥3 h	18	9.4

A majority of adolescents exhibited neutral eating behaviour (93.2%), while only 5.7% reported healthy habits and 1% unhealthy habits. In terms of physical activity, 54.7% were highly active, whereas 44.8% had low physical activity levels, and only 0.5% reported moderate activity.

Screen time varied across domains. For academic use, approximately 51% reported <1 h/day, while smaller proportions reported higher durations. Similarly, for entertainment, gaming, and social media, the majority of adolescents reported screen exposure of less than 1 h/day, although a notable proportion exceeded 2 h across categories.

### 3.3 Association between demographic variables and eating behaviour

The association between demographic variables and eating behaviour is presented in Table 3.

**Table 3:** Association between demographic variable and food habit.

Variable		Unhealthy	Healthy	Chi-square value/likelihood ratio	*p*-value
Sex	Female	87	8	2.523	0.112
	Male	94	3		
Place of living^a^	Hostel	8	0	–	1
	Home	173	11		
Type of family^a^	Nuclear	109	9	–	0.2
	joint	72	2		
Type of food^a^	vegetarian	70	1	–	0.05
	Non-vegetarian	111	10		
Exercise^a^	Yes	108	8	–	0.5
	No	73	3		
Age^a^	12–13	11	0	–	1
	13–14	51	3		
	14–15	119	8		

a Fishers exact test was applied.

The categories were combined because only two adolescents were classified as unhealthy.

No statistically significant association was observed between eating behaviour and most demographic variables, including age, sex, place of living, family type, and exercise status (*p* > 0.05). However, type of food showed a significant association with eating behaviour (*p* = 0.029).

### 3.4 Association between demographic variables and physical activity

The relationship between demographic variables and physical activity is shown in Table 4.

**Table 4:** Association between demographic variable and physical activity.

Variable		Low physical activity	Moderate to physical active	Chi-square value/likelihood ratio	*P*-value
Sex	Female	52	43	7.521	0.006
	Male	34	63		
Place of living^a^	Hostel	3	5	–	0.7
	Home	83	101		
Type of family	Nuclear	61	57	5.900	0.015
	Joint	25	49		
Type of food	vegetarian	35	36	0.924	0.33
	Non -vegetarian	51	70		
Exercise	Yes	44	72	5.578	0.018
	No	42	34		
Age	12–13	11	0	17.758	0.001
	13–14	28	26		
	14–15	47	80		

a Fishers exact test was applied.

Significant associations were observed between physical activity and sex (*p* = 0.006), type of family (*p* = 0.015), exercise status (*p* = 0.018), and age (*p* < 0.05). No significant association was found with place of living and type of food.

### 3.5 Association between demographic variables and screen time

The association between demographic variables and different domains of screen time is presented in Tables 5–9.

**Table 5:** Association between demographic variable and screen time on study.

Variable		<2 h	≥2 h	Chi-square value/likelihood ratio	*P*-value
Sex	Female	83	12	0.319^a^	0.572
	Male	82	15		
Place of living^a^	Hostel	7	1	–	1
	Home	158	26		
Type of family	Nuclear	103	15	0.462	0.497
	Joint	62	12		
Type of food	vegetarian	65	6	2.936	0.087
	Non-vegetarian	100	21		
Exercise	Yes	97	19	0.302	0.254
	No	68	8		
Age^a^	12–13	11	0	–	0.005
	13–14	52	2		
	14–15	102	25		

a Fishers exact test was applied.

**Table 6:** Association between demographic variable and screen time on schoolwork.

Variable		<2 h	≥2 h	Chi-square value/likelihood ratio	*P*-value
Sex	Female	84	11	2.897	0.089
	Male	77	20		
Place of living^a^	Hostel	7	1	–	1
	Home	154	30		
Type of family	Nuclear	100	18	0.180	0.672
	Joint	61	13		
Type of food	vegetarian	62	9	1.002	0.317
	Non-vegetarian	99	22		
Exercise	Yes	96	20	0.260	0.610
	No	65	11		
Age^a^	12–13	7	4	–	0.154
	13–14	47	7		
	14–15	107	20		

a Fishers exact test was applied.

**Table 7:** Association between demographic variable and screen time on entertainment.

Variable		<2 h	≥2 h	Chi-square value/likelihood ratio	*P*-value
Sex	Female	80	15	0.751	0.386
	Male	77	20		
Place of living^a^	Hostel	7	1	–	1
	Home	150	34		
Type of family	Nuclear	99	19	0.930	0.335
	Joint	58	16		
Type of food	vegetarian	58	13	0.000	0.982
	Non-vegetarian	99	22		
Exercise	Yes	93	23	0.502	0.478
	No	64	12		
Age^a^	12–13	9	2	–	0.03
	13–14	50	4		
	14–15	98	29		

a Fishers exact test was applied.

**Table 8:** Association between demographic variable and screen time on game.

Variable		<2 h	≥2 h	Chi-square value/likelihood ratio	*P*-Value
Sex	Female	83	12	0.897	0.344
	Male	80	17		
Place of living^a^	Hostel	7	1	–	1
	Home	156	28		
Type of family	Nuclear	98	20	0.813	0.367
	Joint	65	8		
Type of food	vegetarian	62	9	0.518	0.572
	Non-vegetarian	101	20		
Exercise	Yes	100	16	0.393	0.531
	No	63	13		
Age^a^	12–13	10	1	–	0.9
	13–14	45	9		
	14–15	108	19		

a Fishers exact test was applied.

**Table 9:** Association between demographic variable and screen time on social media.

Variable		<2 h	≥2 h	Chi-square value/likelihood ratio	*P*-value
Sex	Female	78	17	0.679	0.410
	Male	75	22		
Place of living^a^	Hostel	6	2	–	0.66
	Home	147	37		
Type of family	Nuclear	96	22	0.527	0.468
	Joint	57	17		
Type of food	vegetarian	56	15	0.046	0.830
	Non-vegetarian	97	24		
Exercise	Yes	93	23	0.043	0.837
	No	60	16		
Age^a^	12–13	11	0	–	0.18
	13–14	44	10		
	14–15	98	29		

a Fishers exact test was applied.

Across most domains (study, schoolwork, entertainment, gaming, and social media), no significant associations were observed with demographic variables. However, age showed a consistent significant association with screen time in specific domains, including study-related and entertainment screen use (*p* < 0.050).

## 4 Discussion

Adolescence is a critical developmental window during which health behaviours in relation to nutrition, sleep, rest, and exercise are established (12) that the study described eating behaviour, physical activity, and screen time, and examined their distribution across selected demographic variables. Participants were mainly aged 14–15years (66.1%, *n* = 127) with an equal distribution of male (50.51%, *n* = 97) to female (49.5%, *n* = 95) ratio. Majority of adolescents were days scholars with less than 5% of them residing in hostel facilities.

### 4.1 Primary findings

Three main findings emerged regarding eating, activity and screen time. First, most participants reported neutral eating behaviour. Second, just over half were classified as highly physically active, although a proportion reported low physical activity. Third, screen exposure was common across both academic and recreational domains. Further subgroup analysis revealed differences across selected demographic variables, which are discussed below. Dietary routines indicated that three meals per day was the most common pattern (76.0%, *n* = 146), and non-vegetarian food preferences were more prevalent (63.0%, *n* = 121). Although domain specific screen time categories cannot be summed precisely due to overlapping activities, the distributions suggest that adolescents in this cohort may spend approximately 4–6 h per day in cumulative screen exposure across academic and leisure contexts. Mobile phone use was nearly universal (89.6%, *n* = 172), with most adolescents spending 30–60 min daily on mobile devices (62.5%, *n* = 120. Physical activity levels varied with 60.4% (*n* = 116) reported engaging in exercise but 86 of 192 participants self-reporting low physical activity. Of these 52 low vs. 43 moderate were female participants.

### 4.2 Sex vs. physical activity

Association between sex and intensity of physical activity was significant when comparing with male participants (34 low vs. 63 moderate to active). Activity levels comparison between males and females was consistent with 2021 study published in the lancet (13). Significant associations were also observed with family structure, exercise practice and age group suggesting that adolescent movement behaviours are shaped by developmental and household level factors. Adolescent girls in this region often encounter a range of practical and social barriers that limit their participation in structured physical activity (14). In many settings, organised sports or formal exercise may still be viewed as less appropriate for girls due to prevailing sex norms, and concerns about appearance, sweating, or tanning can further discourage engagement. Opportunities for safe and accessible recreation are also frequently limited, with mobility sometimes constrained by safety concerns and fewer school-based facilities or programmes. In addition, academic pressures, household responsibilities, and reduced encouragement from family or peers can leave girls with less time and support to remain active. Together, these internal and external constraints contribute to consistently lower levels of physical activity among adolescent girls, as documented in qualitative work from urban Indian contexts and broader national studies (14, 15, 19). Interestingly, adolescents from joint families were more likely to be classified as moderate-to-active than those from nuclear families (66.2% vs. 48.3%). This finding suggests that household structure may be associated with adolescent physical activity in this cohort. One possible explanation is that joint-family settings may provide social support, supervision, shared responsibilities, or opportunities for informal movement. However, given the cross-sectional design and absence of adjusted analysis, this association should be interpreted cautiously and explored further from a perspective of how wider family ecology impacts health and activity among adolescents.

### 4.3 Dietary patterns among the adolescents

Eating behaviour was assessed in this cohort was as healthy eating, unhealthy eating or neutral. Majority classified as neutral (93.2%, *n* = 179), with healthy habits reported by 5.7% (*n* = 11) and unhealthy habits by 1.0% (*n* = 2). An estimated 149 million children continue to experience stunted growth, while the prevalence of childhood overweight and obesity is increasing across most regions (16). In parallel, suboptimal dietary intake is now recognised as a leading risk factor for mortality (18). Epidemiologically, this may indicate a population in transition, where dietary behaviours are neither strongly protective nor overtly high risk, but may be susceptible to future shifts. In this cohort, meal frequency was largely regular, with 76.0% of adolescents reporting consumption of three meals per day. However, a notable proportion reported less frequent intake, with 17.2% consuming only one or two meals daily, while a smaller subgroup (6.8%) reported eating four times per day. A 27-year prospective cohort study revealed that skipping meals is associated with metabolic syndrome in adulthood (20). Lower meal frequency may reflect insufficient dietary intake, which can contribute to suboptimal nutritional status, reduced cognitive performance and concentration, and the development of unhealthy compensatory eating behaviours later in the day (21). Since the study assessed only the number of meals consumed per day and did not identify which meals were missed, these findings should be interpreted as meal-frequency patterns rather than evidence of specific meal skipping.

### 4.4 Mounting digital exposure

Screen-time patterns were divided across different categories. In each category, nearly half or more of the adolescents reported less than one hour per day, suggesting that individual screen activities were generally moderate. Self-reported screen times and related estimates should be interpreted cautiously. Previous evidence shows that self-reported digital media use deviates from objectively logged use and may be under- or over-reported depending on the screen activity, recall period, and device used (22). Studies report that screen time exceeding two hours per day, as well as eating meals in front of the television, was associated with more than a twofold increase in the likelihood of high consumption of UPF (23). In this study, adolescents reported screen use across both academic and recreational domains. Screen exposure of 2 h or more per day was reported by 14.1% for studying, 16.1% for schoolwork, 18.2% for entertainment, 15.1% for gaming, and 20.3% for social media use. The widespread integration of screens into both learning and leisure contexts suggests an environmental and behavioural shift for young adults (24). Additionally, age demonstrated a statistically significant association with screen exposure, with older adolescents (14–15 years) reporting higher screen time for studying and entertainment, including a greater proportion exceeding two hours per day, highlighting an important target for adolescent focused interventions.

In this study, screen time was not significantly associated with most demographic variables, including sex, family type, type of food, exercise, and place of living. Age was the only variable associated with screen time, and this was observed only for studying and entertainment. This suggests that screen exposure in this cohort may be influenced more by age-related academic and developmental needs than by sex or household factors. The absence of sex differences may also reflect similar access to digital devices among boys and girls, moderate screen durations, and possible school-level limits on recreational screen use.

### 4.5 Demography and its implication on findings

The high prevalence of mobile phone use in this sample shows how rapidly digital media is incorporated in daily routine. Physical activity showed significant associations with sex, family type, exercise status, and age, indicating that it was the behaviour most strongly shaped by demographic factors (14, 15, 19). Interventions must consider that physical activity among adolescents is shaped not only by individual choice but also by household and social context. These interventions should prioritise inclusive and supportive programmes that encourage girls’ participation, such as safe spaces for structured exercise, school-based sports opportunities, and efforts to address sociocultural barriers. An important finding was the difference between the physical activity and exercise responses. This possibly means that adolescents considered everyday activities, such as walking, playing, household work, or active travel, as physical activity, even if they did not do planned or structured exercise. Therefore, the “highly active” category should be interpreted with caution.

This study found that most adolescents had eating-behaviour scores in the intermediate range, while physical activity levels were mainly divided between low and high activity. School-based interventions should move beyond promoting structured exercise alone and create opportunities for everyday movement within school and home environments. Since physical activity varied significantly by sex, family type, exercise status, and age, programmes should be designed to reach adolescents who are less active, with particular attention to inclusive, accessible, and developmentally appropriate activity opportunities.

The presence of meal irregularity among a subset of adolescents also highlights the importance of family and school level nutrition education that promotes consistent, balanced meal routines. Incentivising and providing guidance on healthy digital habits, particularly in relation to academic screen use and sedentary routines must be explored. Age-sensitive screen-time guidance should be prioritised, with practical strategies to help both students and parents support healthy digital engagement across academic and recreational contexts.

#### 4.5.1 Future research

The mismatch between perceived exercise participation and reported structured exercise duration requires further exploration. This may reflect differences in how adolescents understand the terms “exercise” and “physical activity,”. It may also indicate limitations of self-reported physical activity measures, particularly when adolescents are asked to recall both general activity and specific duration. Larger longitudinal studies are needed to examine how these behaviours change over time and interact to influence adolescent health.

Improved measurement precision would strengthen the interpretation of adolescent activity patterns and reduce misclassification arising from varied participant understanding of “exercise” and duration-based reporting. Future research should also adopt longitudinal designs to examine behavioural trajectories over time and clarify causal pathways linking diet, physical activity, and screen engagement.

### 4.6 Strengths

The sample included an almost equal representation of boys and girls, allowing meaningful exploration of sex differences in physical activity. By assessing multiple domains of screen use, including academic and recreational contexts, the study offers a nuanced picture of how digital exposure is integrated into adolescents’ daily routines in the post COVID period (26). The study assessed three interrelated health behaviours using established instruments. The tools were reviewed by five experts and pilot tested among adolescents before the main study, strengthening their clarity, feasibility, and contextual suitability. Assessing academic and recreational screen use separately also provided a more detailed understanding of adolescents’ digital exposure.

### 4.7 Limitations

Several limitations should be considered when interpreting these results. First, the cross-sectional design prevents conclusions about causality or the direction of relationships between eating behaviour, physical activity, and screen time. In addition, the study relied on self-reported measures, which may be affected by recall bias and differences in how adolescents interpret terms such as “exercise” or screen time duration. The AFHC has not been formally validated or culturally adapted for Indian adolescents, and its unhealthy, neutral, and healthy categories were operationally defined for this study rather than externally validated. Although the tools underwent expert review and pilot testing for clarity and feasibility, this did not constitute full psychometric validation in the study population. Objective measures such as accelerometers, detailed dietary assessments, or food diaries were not included. These limitations highlight the need for larger, multi-site longitudinal studies using standardised and objective tools to better capture adolescent lifestyle risk trajectories. The use of purposive sampling may limit the generalisability of findings, as participants were drawn from two selected schools in Mangalore rather than through probability sampling. Findings were reported using significance testing without effect-size measures (e.g., Cramér's V), limiting inference about the magnitude of associations.

## Data Availability

The raw data supporting the conclusions of this article will be made available by the authors, without undue reservation.

## References

[1] Bonnie RJ Backes EP. The Promise of Adolescence: Realizing Opportunity for all Youth. Washington, DC: National Academies Press (2019). 31449373

[2] Viner RM Allen NB Patton GC. “Puberty, developmental processes, and health interventions”. In: Bundy DAP Silva ND Horton S Jamison DT Patton GC, editors. Disease Control Priorities, Third Edition (Volume 8): Child and Adolescent Health and Development. Washington (DC): The International Bank for Reconstruction and Development/The World Bank (2017). p. 107–18. 10.1596/978-1-4648-0423-6_CH9 30212144

[3] Shaw S Muir S Strömmer S Crozier S Cooper C Smith D. The interplay between social and food environments on UK adolescents’ food choices: implications for policy. Health Promot Int. (2023) 38:daad097. 10.1093/HEAPRO/DAAD097 37647523 PMC10468012

[4] Marketing of Ultra-processed and Processed Food and Non-alcoholic Drink Products - PAHO/WHO | Pan American Health Organization. Available online at: https://www.paho.org/en/topics/marketing-ultra-processed-and-processed-food-and-non-alcoholic-drink-products (Accessed June 16, 2026)

[5] Haghjoo P Siri G Soleimani E Farhangi MA Alesaeidi S. Screen time increases overweight and obesity risk among adolescents: a systematic review and dose-response meta-analysis. BMC Prim Care. (2022) 23(1):161. 10.1186/S12875-022-01761-4 35761176 PMC9238177

[6] Phelps NH Singleton RK Zhou B Heap RA Mishra A Bennett JE. Worldwide trends in underweight and obesity from 1990 to 2022: a pooled analysis of 3663 population-representative studies with 222 million children, adolescents, and adults. Lancet. (2024) 403:1027–50. 10.1016/S0140-6736(23)02750-2 38432237 PMC7615769

[7] Ge C Xiong J Zhu R Hong Z He Y. The global burden of high BMI among adolescents between 1990 and 2021. Commun Med. (2025) 5:125. 10.1038/s43856-025-00838-2 40247108 PMC12006325

[8] UNICEF. Poor diets damaging children’s health worldwide, warns UNICEF (2019). Available online at: https://www.unicef.org/eca/press-releases/poor-diets-damaging-childrens-health-worldwide-warns-unicef (Accessed June 16, 2026)

[9] Andreyeva T Kelly IR Harris JL. Exposure to food advertising on television: associations with children’s fast food and soft drink consumption and obesity. Econ Hum Biol. (2011) 9:221–33. 10.1016/J.EHB.2011.02.004 21439918

[10] Geuder Á-JG Martín-María N Geuder Á-JG Martín-María N. Effects of food advertising on youth’s eating behavior: a systematic review of longitudinal studies. Clin Health. (2025) 36:97–108. 10.5093/CLH2025A15

[11] Parajuli J Prangthip P. Adolescent nutrition and health: a critical period for nutritional intervention to prevent long term health consequences. Curr Nutr Rep. (2025) 14(1):116. 10.1007/S13668-025-00706-4 41128775 PMC12549772

[12] Physical activity. Available online at: https://www.who.int/health-topics/physical-activity#tab=tab_1 (Accessed June 16, 2026)

[13] Rakić JG Hamrik Z Dzielska A Felder-Puig R Oja L Bakalár P. A focus on adolescent physical activity, eating behaviours, weight status and body image in Europe, central Asia and Canada: Health Behaviour in School-aged Children international report from the 2021. World Health Organization. Regional Office for Europe (2024). Available online at: https://iris.who.int/handle/10665/376772 (Accessed June 16, 2026)

[14] Ramadass S Gupta SK Nongkynrih B. Adolescent health in urban India. J Family Med Prim Care. (2017) 6:468–76. 10.4103/2249-4863.222047 29416991 PMC5787938

[15] Johnson F Wardle J Griffith J. The adolescent food habits checklist: reliability and validity of a measure of healthy eating behaviour in adolescents. Eur J Clin Nutr. (2002) 56:644–9. 10.1038/SJ.EJCN.1601371 12080404

[16] George LS Jacob A Mohandas KS Ali HM Mathew MM. Level of physical activity among adolescents in Ernakulam district, Kerala: a cross-sectional study. J Family Med Prim Care. (2024) 13:101. 10.4103/JFMPC.JFMPC_907_23 38482286 PMC10931903

[17] Nor Aini J Poh BK Chee WSS. Validity of a children’s physical activity questionnaire (cPAQ) for the study of bone health. Pediatr Int. (2013) 55(2):223–8. 10.1111/ped.12035 23253297

[18] Knebel MTG Da Costa BGG Dos Santos PC De Sousa ACFC Silva KS. The conception, content validation, and test-retest reliability of the questionnaire for screen time of adolescents (QueST). J Pediatr (Rio J). (2021) 98:175. 10.1016/J.JPED.2021.05.004 34174211 PMC9432249

[19] Lyngdoh M Akoijam BS Agui RS Sonarjit Singh K. Diet, physical activity, and screen time among school students in Manipur. Indian J Community Med. (2019) 44:134. 10.4103/IJCM.IJCM_282_18 31333291 PMC6625269

[20] Wennberg M Gustafsson PE Wennberg P Hammarström A. Irregular eating of meals in adolescence and the metabolic syndrome in adulthood: results from a 27-year prospective cohort. Public Health Nutr. (2016) 19(4):667–73. 10.1017/S1368980015001445 25936413 PMC10271199

[21] Paoli A Tinsley G Bianco A Moro T. The influence of meal frequency and timing on health in humans: the role of fasting. Nutrients. (2019) 11(4):719. 10.3390/nu11040719 30925707 PMC6520689

[22] Zhao Y Han X Bagot KS Tapert SF Potenza MN Paulus MP. Examining measurement discrepancies in adolescent screen media activity with insights from the ABCD study. NPJ Ment Health Res. (2025) 4(1):15. 10.1038/s44184-025-00131-z 40346141 PMC12064680

[23] Carducci B Chen ZH Campisi SC Miliku K. Adolescence as a key developmental window for nutrition promotion and cardiometabolic disease prevention. NPJ Metab Health Dis. (2025) 3:40. 10.1038/s44324-025-00082-1 41073489 PMC12514207

[24] van Sluijs EMF Ekelund U Crochemore-Silva I Guthold R Ha A Lubans D. Physical activity behaviours in adolescence: current evidence and opportunities for intervention. Lancet. (2021) 398:429–42. 10.1016/S0140-6736(21)01259-9 34302767 PMC7612669

[25] Rodríguez-Barniol M Pujol-Busquets G Bach-Faig A. Screen time use and ultra-processed food consumption in adolescents: a focus group qualitative study. J Acad Nutr Diet. (2024) 124:1336–46. 10.1016/j.jand.2024.04.015 38697354

[26] Resende MAA Da Fonseca ML De Freitas JT Gesteira E Rossato LM. Impacts caused by the use of screens during the COVID-19 pandemic in children and adolescents: an integrative review. Rev Paul Pediatr. (2024) 42:e2022181. 10.1590/1984-0462/2024/42/2022181

